# Drivers of longevity of wild-caught *Aedes albopictus* populations

**DOI:** 10.1186/s13071-023-05961-4

**Published:** 2023-09-16

**Authors:** Laura Blanco-Sierra, Simone Mariani, Santi Escartin, Roger Eritja, John R. B. Palmer, Frederic Bartumeus

**Affiliations:** 1 Centre d’Estudis Avançats de Blanes (CEAB-CSIC), Girona, Spain; 2 CREAF, Cerdanyola del Vallès, Spain; 3 ICREA, Institució Catalana de Recerca i Estudis Avançats, Barcelona, Spain; 4 Universitat Pompeu Fabra, Barcelona, Spain

**Keywords:** *Aedes albopictus*, Demography, Frailty, Seasonality, Survival, Vector-borne disease risk

## Abstract

**Background:**

Age structure and longevity constitute fundamental determinants of mosquito populations’ capacity to transmit pathogens. However, investigations on mosquito-borne diseases primarily focus on aspects such as abundance or dispersal rather than survival and demography. Here, we examine the post-capture longevity of wild-caught populations of the Asian tiger mosquito *Aedes albopictus* to investigate the influence of environmental factors and individual frailty on longevity.

**Methods:**

We captured females of *Ae. albopictus* from June to November 2021 in a vegetated and an urban area by two methods of capture (BG traps and Human Landing catch). They were kept in semi-controlled conditions in the field, and survival was monitored daily across the 859 individuals captured. We studied the differences in longevity per capture method and location and the influence on longevity of seasonal, climatic and individual factors.

**Results:**

Photoperiod, GDD, minimum and maximum temperature and relative humidity showed an effect on the risk of death of females in the field. Females captured in urban area with Human Landing catch methods had greater longevity than females captured in non-urban areas with BG traps. Individual variance, reflecting individual frailties, had an important effect on the risk of death: the greater the frailty, the shorter the post-capture longevity. Overall, longevity is affected not only by climate and seasonal drivers like temperature and photoperiod but also by the individual frailty of mosquitoes.

**Conclusion:**

This work unravels environmental drivers of key demographic parameters such as longevity, as modulated by individual frailty, in disease vectors with strong seasonal dynamics. Further demographic understanding of disease vectors in the wild is needed to adopt new surveillance and control strategies and improve our understanding of disease risk and spread.

**Supplementary Information:**

The online version contains supplementary material available at 10.1186/s13071-023-05961-4.

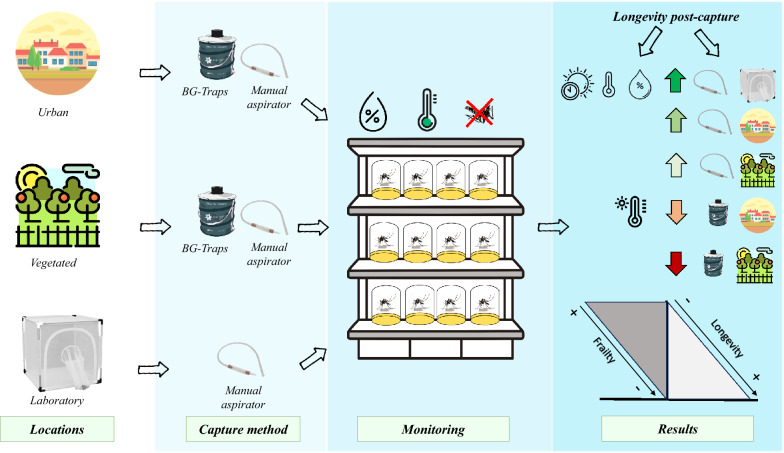

## Background

Demography is concerned with the size, distribution, structure and dynamics of populations [1]. Understanding the determinants of demographic patterns in wild animal populations is not only crucial for biological conservation and management [2, 3] but is also important for studies of epidemiology [4].

Along with population dynamics, knowledge about the age structure and longevity of disease vectors, such as mosquitoes, is crucial to understanding how diseases spread [5]. Pathogens transmitted by mosquitoes generally require time to replicate and spread in the mosquito’s salivary glands. Only if the mosquito survives longer than the extrinsic incubation period (the interval of time between the ingestion of pathogen infected blood from one host and the transmission of the pathogen by biting another host) can it succeed in transmitting the pathogen [6]. In fact, adult survival is one of the key parameters in MacDonalds’s classic equation for disease transmission risk [4], and behavioural and physiological adaptive strategies have allowed mosquitoes to extend their longevity [7], and consequently the overall risk of disease transmission.

Given this influential role of survival in the transmission of mosquito-borne diseases, knowledge of the extrinsic and intrinsic factors that can limit the life expectancy of these insects is essential. Seasonality and weather variation play a critical role in mosquito population dynamics, with temperature being the most important extrinsic factor [8]. The effect of humidity in survival is not as clear. While some laboratory work suggests that humidity is not a limiting factor for mosquito survival [9], others show reduced survival at low relative humidity levels [10].

Frailty, the condition of being weak and more vulnerable to death, also affects the overall longevity of individuals. Frailty is often understood as an intrinsic component of survival that can vary with genotype and age, but also depends on the phenotype and the overall life condition (e.g. accumulation of metabolic stressors that decrease the chances of survival). From a demographic perspective, individual frailty is strongly associated with age; among adult mosquitoes, young individuals have the greatest reproductive potential and fewest mortality risks, while older individuals have lower reproductive potential and higher mortality risks [11].

Despite the epidemiological importance of age in disease-transmitting species and current age-grading tools [12], there is no standardised methodology available to estimate the age structure of insect populations in an efficient and inexpensive way [11]. Some of the methods used at present, such as transcriptional profiling [6], the use of follicular relics [13] or near-infrared spectroscopy (NIRS) [14], can distinguish young individuals from old ones, but they are not accurate enough to classify mid- and advanced-aged individuals [11]. Others, such as mark-release and recapture, are labour intensive and could overestimate mortality by losses during emigration or removal sampling [15].

With the objective of finding a way to infer longevity and estimate age structure in wild insect populations, Carey, Müller and colleagues [16] developed the captive cohort method (CCM) as a demographic technique. In this approach, the distribution of remaining longevities for wild-caught individuals can be used to infer the age structure dynamics of wild populations. Similar to natural lifespans, post-capture longevity of wild-captured mosquitoes are conditioned by: (i) their environment and (ii) their physiological and chronological ages [17]. Differences between the pre- and post-capture conditions include the absence of certain environmental hazards (e.g. predation), which can impact the potential longevity.

Here, we apply the CCM in the field to assess the factors influencing post-capture longevity in mosquito populations, both extrinsic (e.g. temperature, photoperiod, humidity) and intrinsic (individual frailty). Our aim is to understand how these factors affect mosquito population dynamics and promote additional effort in the study of demography and survival of insects in the wild, key to understanding the dispersal mechanisms and the success in the transmission of disease-causing pathogens.

Our target species is the Asian tiger mosquito  *Aedes albopictus*. This highly invasive species, originated in Southeast Asia [18], has colonized Africa, Europe and America (north and south) in recent decades, after first extending its range eastwards across the Pacific islands during the early twentieth century [19]. This ease of expansion poses a serious risk to human health because of the species’ capability of transmitting several pathogens. In laboratory conditions, *Ae. albopictus* can transmit more than 20 arboviruses and, in the wild, it has shown competence as a vector of dengue and chikungunya viruses, among others [20].

## Methods

### Field sampling

**Fig. 1:**
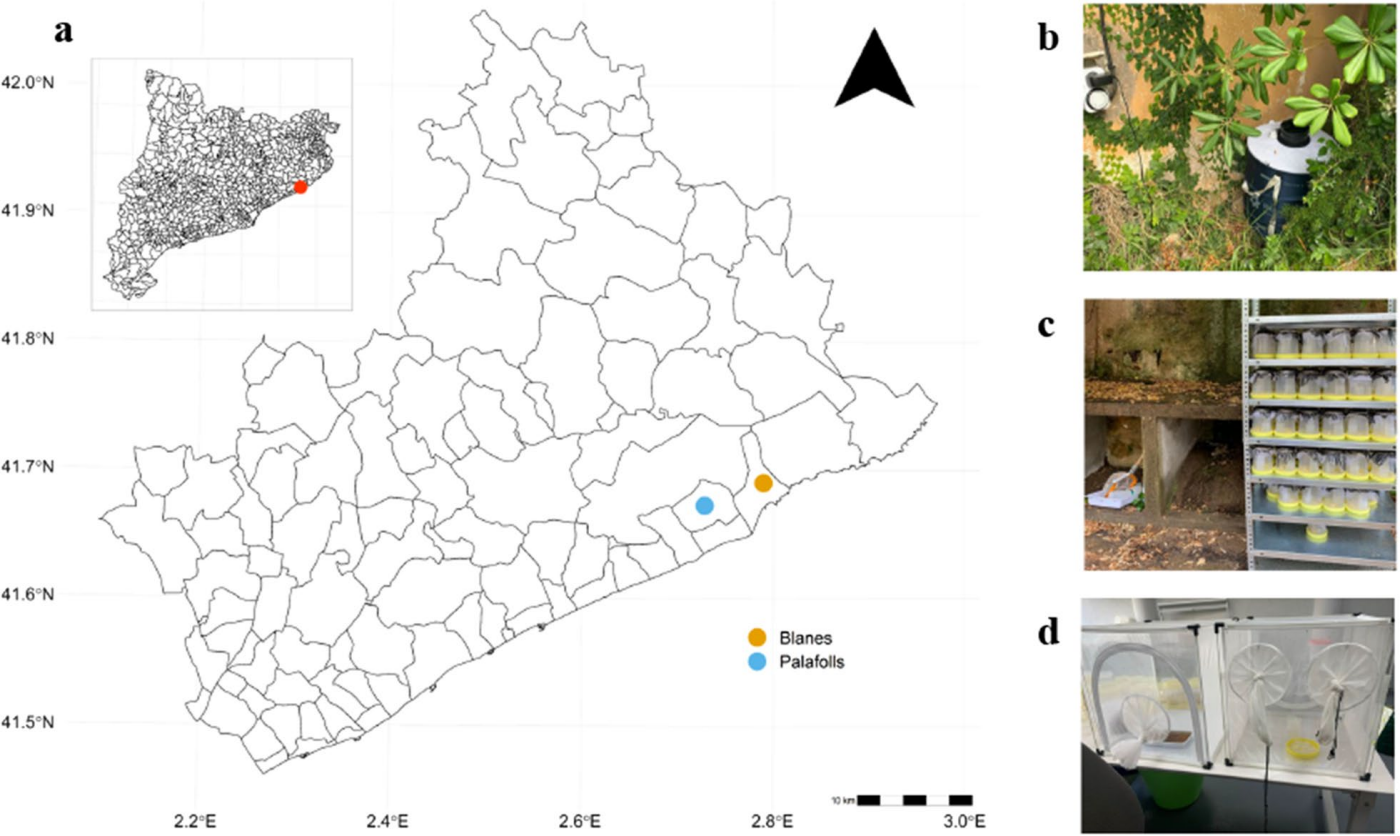
**A** Map of Catalonia, Spain (top left) and study area (center); red point in top left map indicates the southern coast of the province of Girona; in the study area map blue point indicates Palafolls (urban) and yellow indicates Blanes (vegetated); **B** BG-Sentinel trap in the vegetated location, with timer installed. **C** Shelves installed in the field and laboratory locations, with the individual containers for the female mosquitoes. **D** Cages for raising colonies in the laboratory

From June to November 2021, we sampled adult female *Aedes albopictus* mosquitoes daily in Catalonia, Spain. The study locations included an urban area in Palafolls (41.669167$^\circ$N, 2.750556$^\circ$E) within the Maresme region, approximately 68 km east of Barcelona, and the Jardí Botanic Marimurtra, a botanical garden located in Blanes (41.676667$^\circ$N, 2.801944$^\circ$E), 5 km east of Palafolls (Fig. 1a). Both sites offered suitable breeding sites for *Ae. albopictus*, with some variations between the urban and vegetated areas. The urban site, situated downtown in Palafolls with a population of approximately 10,000 residents, had numerous houses with gardens and various public and private water irrigation systems. In contrast, the vegetated site had a smaller residential population in the surroundings but experienced a high influx of tourists, providing a great availability of hosts. The presence of vegetation and continuous irrigation in the area created ample mosquito breeding sites and refuges, resulting in a more humid and shaded environment.

Mosquitoes were captured using two methods: BG-Sentinel traps (BGT) with BG-Lure attractant and Human Landing catch (HLC) with a handheld mouth aspirator. Upon capture, each mosquito was immediately transferred to an individual container labeled with an ID code, location, capture method and date of capture. BGTs (three in the Jardí Botanic Marimurtra and one in Palafolls) were checked daily, collecting only females of *Ae. albopictus*, with an average of $\approx$ 5 individuals per day (ranging from 1 to 10 females depending on the moment of the season and mosquito presence). To minimize potential negative impacts, the BGTs were active during specific periods corresponding to increased *Ae. albopictus* activity [21, 22], controlling the fan with timers for two intervals of approximately 4 h each in the morning and before sunset to prevent dehydration caused by continuous airflow exposure (Fig. 1b). In addition to BGTs, we used HLC to capture females. This involved capturing mosquitoes as they landed on the collector while checking the BGTs, transferring females to individual containers and monitoring mortality ($\approx$ 20 min). Females captured by HLC followed the same protocol as the BGT-captured females, as described below.

Individual cages, comprising transparent plastic containers (1000 ml) with a side opening covered by mosquito net, were used for each mosquito. Inside the containers, females were provided with a 10% sugar solution for nourishment. We replenished the sugar solution in each individual cage as needed to maintain food quality and prevent fungal contamination. All individual containers in both locations were placed on a shaded shelf with six levels (Fig. 1c). Temperature and relative humidity were continuously recorded using HOBO$^\circledR$ MX1101 data loggers. Each mosquito was checked daily at the same time (±1 h), and deaths were noted and removed.

### Laboratory

The laboratory populations of *Ae. albopictus* came from eggs collected at the Jardí Botanic Marimurtra every week from June to October by means of six ovitraps placed around the garden.

Immature stages were fed increasing amounts of fish food as they grew (from $\approx$ 0.1 mg of flakes/larva/day in L1 to $\approx$0.4 mg/larva/day in L4) [23]. Thirteen cohort cages of $\approx$120 mosquitoes were established with predominance of females over males (ratio 2:1), maintaining larvae and adults under semi-controlled climatic conditions (22–25 °C; $\sim$1 °C daily fluctuations; 60–70%RH, natural cycle L:D) (Fig. 1d). Daily samples of $\approx$ 10 females of known ages ranging from 2 to 27 days from each cohort cage were transferred to individual containers with a handheld mouth aspirator and monitored. Containers were labelled with mosquito ID, colony start date and capture date. Individuals were inspected daily, and dead mosquitoes were noted and removed.

### Statistical analysis

#### Post-capture longevity: mean difference tests

We examined the influence of location (urban and vegetated) and capture method (BGT and HLC) on remaining longevity after capturing females of unknown age in the field. We performed a Shapiro-Wilk test (*W* = 0.95, *P* < 0.0001) and graphical methods (Additional file 1: Fig. S1) to determine if our longevity data followed a normal distribution. Due to the non-normal distribution of the data we compared the differences between mean survival rates with Wilcoxon-Mann–Whitney and Kruskal-Wallis tests. Considering varying sample sizes (HLC: N = 157, BGT: N = 702), we also conducted bootstrap analyses [24]. These involved comparing N individuals from each location (where N represents the minimum sample size obtained for the HLC condition) with N individuals randomly sampled from the BGT captures. We iterated this comparison 999 times and then computed a pseudo *P*-value counting how many times these comparisons were statistically significant.

#### Post-capture survival modelling: Kaplan-Meier and Cox regressions

We estimated survival functions for three location groups (urban, vegetated and laboratory) and for each method of capture (BGT and HLC) in each field location. The Kaplan-Meier estimator was used considering escaped or artificially killed mosquitoes as censored observations. We performed pair-wise comparisons among the different locations and methods with the log-rank test using the *survival* package [25] for R version 4.2.0 [26].

We performed a Cox regression analysis to assess the influence of environmental factors on post-capture survival. Hazard ratios (HR) and 95% confidence intervals (95% CI) were calculated to determine the impact and significance of each variable. We used daily temperature and humidity measurements from our HOBOs and computed the Growing Degree Day (GDD), a measure of heat accumulation used to predict plant and animal development rates (e.g. the date in which an insect will emerge from dormancy). As seen in Delatte et al. [27], the lower developmental temperature was found at 10.4 °C, with an optimum of 29.7 °C. Therefore, GDDs were calculated with a Tbase of 10 °C and a maximum temperature limit of 30 °C. Two additional variables, GDD per captured female (*gdd_id*; value of heat accumulation for each female during its captivity period to death) and daily differences in GDD (*daily_acc_gdd*), were computed. The *pollen* package [28] in R version 4.2.0 [26] was employed for GDD calculations. Photoperiod (hours of light/day) was calculated for the entire experimental period with the *meteor* package [29] in R version 4.2.0 [26] and used as covariable. Since survival was dependent on location and capture method, both variables were treated as levels. A simple Cox regression model examined the impact of location and capture method on survival, while a time-dependent mixed Cox regression model incorporated daily changing environmental variables [30, 31]. To account for clustered variance due to multiple events observed daily for each mosquito, an individual mosquito level was included in the analysis.

Potential correlation (see Additional file 1: Fig. S2) and collinearity were assessed using the *corrplot* [32] and *car* [33] packages for R version 4.2.0 [26], respectively. Model selection was based on the Akaike information criterion (AIC). Cox models were implemented using the *survival* [25] and *coxme* [34] packages in R version 4.2.0 [26].

## Results

### Post-capture longevity: raw data and mean comparisons

**Fig. 2:**
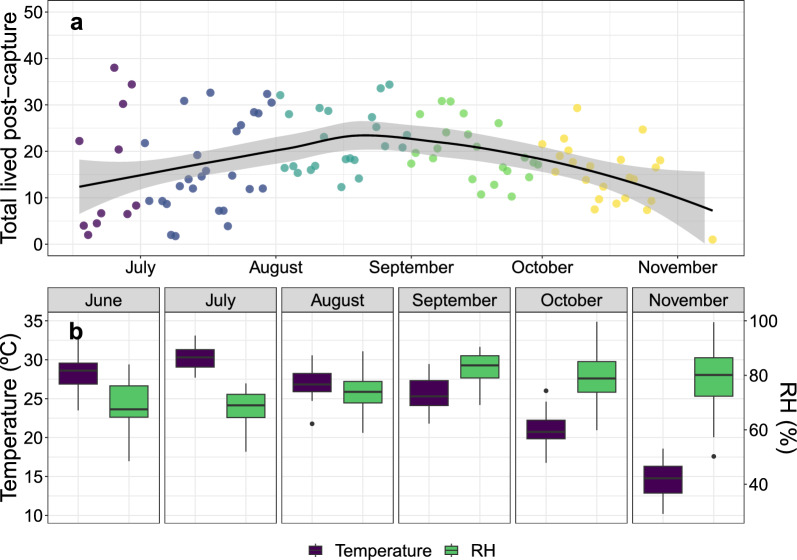
Post-capture longevity of female *Aedes albopictus* captured in both study sites from June to November 2021. **A** Points represent the average post-capture longevities of individuals sampled each day. The black line represents the trend of longevity through the season with its 95% confidence interval. **B** Box plots of the average daily temperature (°C) and relative humidity (%) values grouped by month during the experimental season

A total of 859 *Ae. albopictus* females were monitored over a 168-day period between 18 June and 3 December 2021 (death of the last female) (Fig. 2). The post-capture longevities of female *Ae. albopictus* mosquitoes significantly differed based on capture methods (BGT vs. HLC), locations (urban area vs. vegetated area) and capture methods within each location, suggesting variations in the potential longevity of females among the compared groups. In the urban location, the mean post-capture longevity of *Ae. albopictus* females was 19.33 days (SD 16.37), with a range of 1 to 86 days. In the vegetated location, the mean post-capture longevity was 15.94 days (SD 14.41), ranging from 1 to 74 days. We utilized bootstrapping methods to examine differences in post-capture longevity according to capture method within each location. On average, females captured with BG traps had significantly lower post-capture longevity, with mean values of 17.31 days (SD 15.60) in the urban area and 14.63 days (SD 13.51) in the vegetated area. However, those females captured with human landing catch (HLC) showed better mean post-capture longevity values; 28.34 days (SD 16.75) in the urban area and 21.84 days (SD 16.79) in the vegetated area (Table 1).

**Table 1:** Differences in post-capture longevity of females captured in both urban and vegetated field locations and by both capture methods

Groups	Mean ± SD	Range (min–max)
Field locations*		
Urban	19.33±16.37$^a$	1–86
Vegetated	15.94±14.41$^b$	1–74
Capture methods**		
HLC	25.11±17.03$^a$	1–84
BGT	15.97±14.67$^b$	1–86
Location + Method field***		
Vegetated - BGT	14.63±13.51$^a$	1–69
Vegetated - HLC	21.84±16.79$^b{^c}$	1–74
Urban - BGT	17.31±15.60$^a{^b}$	1–86
Urban - HLC	28.34±16.75$^c$	1–84

*Different superscripts indicate significant differences according to the Wilcoxon-Mann-Whitney test (*P* < 0.05)

**Different superscripts indicate significant differences according to the Wilcoxon-Mann-Whitney test (*P* < 0.05)

***Different superscripts indicate significant differences according to the post hoc Dunn test (*P* < 0.05) after performing a Kruskal-Wallis test (*P* < 0.05)

Pooling the data from both field locations, we observed a seasonal pattern on post-capture longevity (Fig. 2). In both the urban and vegetated locations, post-capture longevity was lower during June and July: 12.67 days (SD 13.42) in the urban site; 14.22 days (SD 13.40) in the vegetated site) compared to August and September (23.66 days (SD 17.43) in the urban site; 17.69 days (SD 15.49) in the vegetated site. For October and November the mean post-capture longevity was also lower than in August and September (16.21 days with SD of 13.20 in urban; 12.29 days with SD of 10.82 in vegetated). In the urban area, the post-capture longevity in June and July differed significantly from both August and September. September and October in the urban area also revealed significant differences. In the vegetated area, significant differences were found only between August and October (for detailed information, see Additional file 1: Figs. S3–S4).

Noticeably, the maximum temperatures recorded in both field locations never exceeded 33.1 °C. Indeed, the 30 °C threshold was rarely surpassed, except for occasional temperature peaks in July and August. Consequently, the females were not exposed to extreme temperatures. However, significant differences in temperature variations were observed among the locations (see Additional file 1: Fig. S5). The values of relative humidity (RH%) were generally high; on average, the maximum average values of RH% were 92.42% (SD ± 8.75) in the vegetated area and 87.61% (SD ± 7.05) in the urban area.

### Survival curves: influence of capture method, location and seasonality

**Fig. 3:**
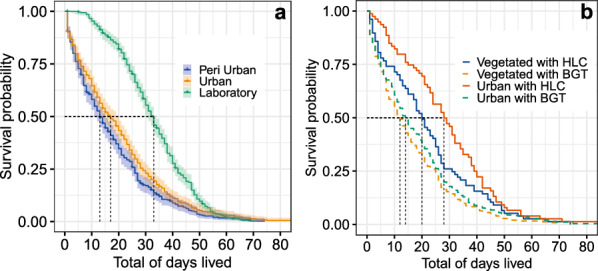
Kaplan-Meier curves showing cumulative survival probabilities as a function of post-capture days lived for female *Aedes albopictus*: **A** captured in field and laboratory locations; **B** captured in each field location and with each capture method

**Fig. 4:**
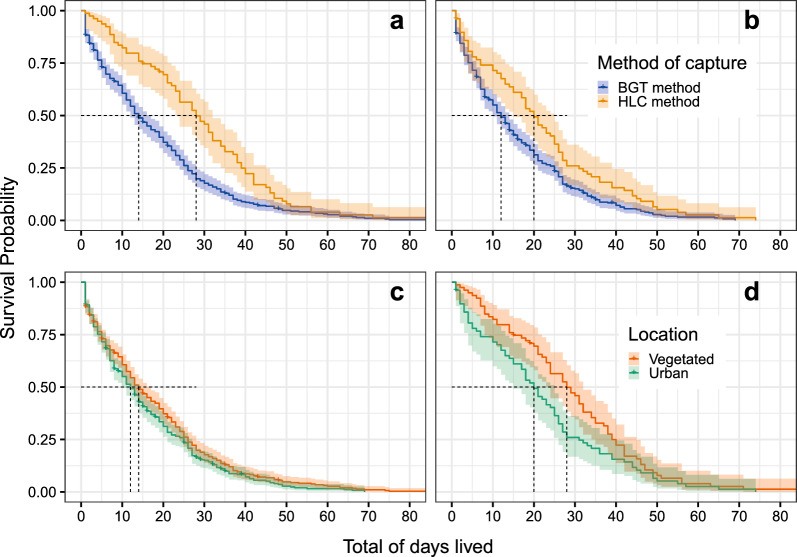
Survival curve comparison across methods of capture and location of females in the field showing cumulative survival probabilities as a function of post-capture days lived for female *Ae. albopictus*: **A** in the vegetated location by each method of capture; **B** in the urban location by each method of capture; **C** captured by BGT in each field location; **D** captured by HLC in each field location

The Kaplan-Meier estimator [35] and pairwise comparisons among the survival rates clearly reproduced the differences between the post-capture survival curves in the field and in the laboratory (Fig. 3a). Females raised in the laboratory showed a higher survival rate than the females captured and kept in the field (*P* < 0.0001). If we compare the survival rates of the field groups, we observe that females captured in the urban area with HLC had the highest survival rate, while females captured in the vegetated area with BGT had the lowest survival rate (Fig. 3b; *P* < 0.0001). In general, HLC was associated with greater longevity compared to BGT, whatever the location (Fig. 4a, b), and the survival rate in the urban area was slightly higher than that of the females from the vegetated area, whatever the capture method (Fig. 4c, d). For more details, see Additional file 1: Table S1.

### Cox regression analysis

Cox regression analysis revealed location and capture method as significant predictors of the risk of death. Urban areas showed lower risk of death (HR = 0.837; 95% CI = 0.72$-$0.96; *P* < 0.05) than vegetated areas. The same analysis revealed BGT (HR = 1.564; 95% CI 1.31$-$1.86; *P* < 0.0001) as a significant predictor of an increased risk of death compared with HLC method.

Mixed effect Cox regression models showed that larger photoperiod values (hours of light/day) decreased the risk of death (HR = 0.941; 95% CI = 0.89$-$0.99; *P* < 0.05), while more growing degree days computed at the individual level (*gdd_id* ) increased the risk of death (HR = 1.0005; 95% CI = 1.0001 $-$ 1.001; *P* < 0.01). When both *gdd_id* and photoperiod were combined in the same model, they maintained their influence. Adding maximum relative humidity to photoperiod and *gdd_id* revealed maximum relative humidity as a strong predictor related with lower risk of death (HR = 0.984; 95% CI = 0.97 $-$ 0.99; *P* < 0.0001).

**Table 2:** Summary of the Mixed effects Cox regression models

*Fixed effects*	*Model 1*	*P*	*Model 2*	*P*	*Model 3*	*P*	*Model 4*	*P*	*Model 5*	*P*	*Model 6*	
	HR		HR		HR		HR		HR			
	(95% CI)		(95% CI)		(95% CI)		(95% CI)		(95% CI)		(95% CI)	
Photoperiod	0.941	*			0.947	*			0.931	**		
	(0.89-0.99)				(0.89-0.99)				(0.88-0.98)			
GDD (id)			1.0005	**	1.0001	**			1.0005	**		
			(1.0001-1.001)		(1.0001-1.001)				(1.0001-1.001)			
TMax							0.979	**				
							(0.96-0.99)					
Tmin											0.967	***
											(0.95-0.98)	
RHmin											0.989	***
											(0.98-0.99)	
RHmax							0.985	***	0.984	***		
							(0.98-0.99)		(0.97-0.99)			
*Random effects*	$\sigma ^2$		$\sigma ^2$		$\sigma ^2$		$\sigma ^2$		$\sigma ^2$		$\sigma ^2$	
*Location*$^1$	0.011		0.010		0.012		0.019		0.031		0.058	
*Method*$^2$	0.084		0.092		0.093		0.085		0.098		0.084	
*Id_mosquito*	8.2e-05		2e-04		3e-04		4.1e-05		3e-04		3e-05	
**AIC**	18.48		20.71		22.64		29.58		36.80		44.20	

$$^*\ \textit{P} < 0.05$$

$$^{**}\ \textit{P} < 0.01$$

$$^{***}\ \textit{P} < 0.0001$$

^1^ Vegetated and urban areas

^2^ Human Landing catch and BG traps

The average daily maximum values of temperature (HR = 0.979; 95% CI = 0.96 $-$ 0.99; *P* = 0.01) and humidity (HR = 0.985; 95% CI = 0.97$-$0.99; *P* < 0.001) decreased the risk of death significantly. Results were the same, i.e. the larger the values, the lower the risk of death, when examining the minimum daily values of temperature and humidity (for more details, see Table 2).

### Influence of the individual random effects in the post-capture longevity

**Fig. 5:**
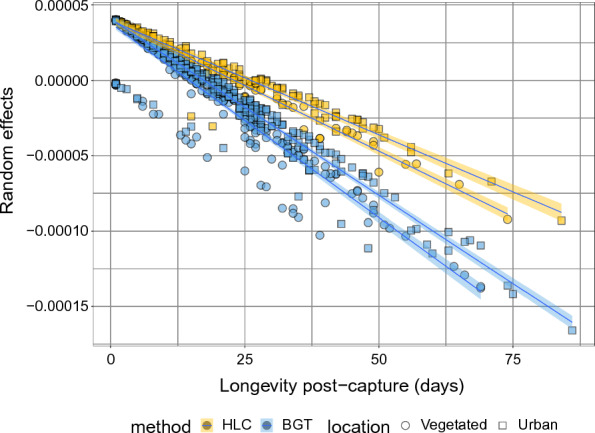
Individual variation of mixed effect Cox regression models capturing age/frailty effects on longevity. Individual level variation in random intercepts from Model 4 (see Table 2) against post-capture longevity, for the four different data pools (Urban-BGT; Urban-HLC; Vegetated-BGT; Vegetated-HLC)

To assess individual variability in post-capture longevity, we extracted random effects from the Cox mixed models by incorporating a variable (level) with a unique ID value for each individual mosquito. The intercepts of this variable represent individual variation in survival that could be associated to individual age or frailty. Figure 5 illustrates the negative correlation between these intercept values (frailty/age) and longevity. Mosquitoes captured with BGTs showed a stronger negative correlation between such random intercepts and post-capture longevity than mosquitoes captured with HLC, suggesting larger individual frailty/age in association with the BGT capture method. The differences observed among location (urban vs. vegetated) were minor, with vegetated areas (circles) showing slightly stronger negative slopes than urban areas (squares) for both capture methods. HLC-captured mosquitoes in the urban area displayed the lowest negative relationship between frailty/age and longevity post-capture.

## Discussion

During part of the twentieth century, mosquito studies in vector ecology assumed that mosquito mortality remained constant across ages [4]. This assumption suggested that all mosquitoes had the same potential for disease transmission regardless of age. This would apply to various determinants of disease transmission, including biting rate, host preference and dispersal capacity [36]. However, it is now widely recognized that mosquitoes do undergo senescence and that age and longevity play a crucial role in their ability to transmit pathogens [36, 37]. In this work, we followed the captive cohort method (CCM) [16] to gather unique data on the longevity potential of wild-caught *Ae. albopictus* populations. By assessing post-capture longevity under semi-controlled conditions, we aimed to investigate the determinants of longevity, including extrinsic factors (e.g. temperature, humidity, photoperiod) and the role of individual frailty. Although we were unable to measure other hazards (e.g. predation in the wild), our results reveal clear seasonal patterns in the potential longevity of *Ae. albopictus* populations, placing the peak of survival of previously captured mosquitoes between late July and early September. These observations coincide with the abundance peaks observed in other studies on the activity and population dynamics of *Ae. albopictus* in Catalonia, Spain [38].

The method by which we captured mosquitoes significantly influenced longevity estimates. Females captured with HLC exhibited higher survival rates compared to BGTs, despite making efforts to minimize damage and stress caused by the fan in the BGTs (see Methods section). Hence, we still observed a negative effect on post-capture longevity by BGTs likely because of the stronger suction of the fan compared to manual aspiration.

Local habitats appear to significantly impact mosquito survival, regardless of the capture method. Urban areas showed larger *Ae. albopictus* survival rates compared to vegetated locations. Different habitats provide different resources and environmental stressors, resulting in diverse post-capture longevities. Although it was previously considered a rural vector [39], *Ae. albopictus* has successfully adapted to urban environments, with more availability to breed in artificial containers [40, 41]. Urban areas tend to exhibit more larval habitat availability, shorter development time, increased emergence rates and extended longevity [41]. In our study, both field sampling sites had ample breeding sites, but control measures at the Jardí Botànic Marimurtra (e.g. emptying pots with accumulation of water from rains or irrigation, use of biological larvicides four times between April and October at the main known breeding sites, removal of garden bromeliads) likely slowed mosquito population growth. Therefore, we may expect young individuals to be under represented in the vegetated area, leading to aged populations. The latter could explain the reduced post-capture longevity observed in the vegetated compared to the urban site. In addition, laboratory-reared females exhibited higher survival rates compared to those collected from urban and vegetated locations. This difference is likely due to reduced stress due to gentle transfers to individual cages in the laboratory (i.e. handheld mouth aspirator) and the subsequent environmental steadiness in the cages as well as a prevalence of young females, i.e. a wide age range of laboratory experimental subjects (2–27 days) compared to field experimental subjects, where young females are expected to be less represented.

The post-capture longevity of wild-caught female mosquitoes exhibited a clear seasonal pattern, with lower average lifespan during spring and autumn compared to summer months. In summer, wild populations are expected to be younger and so post-capture longevity becomes longer. This pattern is influenced by external factors such as temperature and photoperiod [8]. Our study reveals that growing degree days, photoperiod and maximum/minimum temperature and humidity strongly influence post-capture longevity. Mosquitoes, being poikilothermic organisms, are susceptible to temperature variations that impact their body temperature [42]. Temperature plays a crucial role in the population ecology of *Ae. albopictus*, as highlighted by previous studies [27, 43, 44]. Highest adult survival was observed at 15 °C, while the lowest was found at 30–35 °C. Other studies reported the range of 25–30 °C as suitable for them to live, but higher temperatures suggested a decline in life expectancy in laboratory conditions [45–47]. *Ae. albopictus* has adapted well to mild temperatures, enabling its establishment in temperate regions [27]. The moderate temperature values within the experimental areas did not have a negative impact on post-capture longevity. However, we showed that increasing heat accumulation (measured as growing degree days) had a negative impact on mosquito survival. At our study sites, relative humidity (RH%) showed a stable seasonal pattern (Additional file 1: Fig. S5 and Results). Previous works have shown that *Ae. albopictus* thrives in RH% ranges of 60–90% with minimal impact on adult survival [27, 47]. However, other studies showed that RH% values of 35% and 70%, not usual in our data, negatively affected survival [10, 48]. Overall, our results support a reduced risk of death (HR < 1) with increasing RH%. Photoperiod encompass various seasonal changes and can serve as a cue for changes in insect development, growth and behaviour. For certain mosquito species, such as *Ae. albopictus*, photoperiod can also act as a signal to induce diapause as unfavourable conditions approach [49]. Our findings indicate a significant association between more daylight hours per day and decreased risk of death. While other studies have shown that shorter days prompt mosquitoes to initiate different strategies for coping with cold conditions, such as larger body size or diapause, it has not been demonstrated that *Ae. albopictus* females increase their longevity with shorter day lengths [9, 49, 50].

In addition to environmental factors, which resulted in a clear seasonal pattern with higher post-capture longevity in August and September and lower at the beginning and end of the mosquito season, we observed significant individual variation in post-capture longevity. We hypothesize that individual variability in post-capture longevity may reflect inherent variation in frailty, which in turn is associated with aging, genetics or phenotypic degradation resulting from previous hazards encountered before capture. Our results demonstrated that females with shorter post-capture longevity exhibited greater frailty compared to those with longer post-capture longevity. The age distribution of the *Ae. albopictus* population may have changed over the season, and daily captures could have allowed us to observe these age-related differences over time. These findings align with Carey’s theory [1], which states that the shorter an individual lives after capture, the older it probably was when captured.

## Conclusion

Acknowledging the limitations of our methods and recognizing that our estimations do not represent true survival curves, it is clear that our post-capture survival estimations effectively capture the natural individual variability and seasonal patterns influenced by changing environmental conditions. Our findings highlight the complex interplay between environmental and intrinsic drivers of longevity, deserving further research. We believe that understanding mosquito demographic patterns in the wild is essential for deploying more comprehensive mosquito surveillance and control strategies.

### Supplementary Information

**Additional file 1: Table S1.** Kaplan-Meier analysis, comparing survival curves of differenr locations (field and labpratory), capture methods (human landing catches, BG-Sentinel trapping) and their combinations.** Figure S1.** Histogram and Shapiro-Wilk test to assess normality of the longevity data. **Figure S2.** Correlation plot of the variables included in the Cox mixed models. **Figure S3.** Kruskall-Wallis and Dunn test with Bonferroni correction, showing the differences in post-capture longevity through the months of the experiment in the urban area. **Figure S4.** Kruskall-Wallis and Dunn test with Bonferroni correction, showing the differences in post-capture longevity through the months of the experiment in the vegetated area. **Figure S5.** Significance test for the differences between the meteorological conditions of (a) temperature and (b) relative humidity (b) both field sites.

## Data Availability

The dataset used and the codes to carry out the analysis and graphs are available in the following GitHub repository: https://github.com/lblancozgz/driverslongevity
