# Supplementary material for: Drivers of longevity of wild-caught Aedes albopictus populations
Source: Parasit Vectors. 2023 Sep 16;16:328. doi: 10.1186/s13071-023-05961-4 (PMC10504710; doi:10.1186/s13071-023-05961-4)

Table S1. Kaplan Meier analysis, comparing survival curves of different locations (field and laboratory), capture methods (Human landing catches, BG-Sentinel trapping), and their combinations.

Groups	n	median	CI95%
Field locations			
Urban	430	17	14-20
Vegetated	429	13	12-15
Log-rank test (p)		0.01	
Experimental locations			
Urban	430	17	14-20
Vegetated	429	13	12-15
Laboratory	491	33	31-34
Log-rank test (p)		<2e-16	
Capture methods*			
BGT	702	13	12-14
HLC	157	24	21-27
Log-rank test (p)		4e-07	
Field location + Method capture			
Vegetated - BGT	351	12	10-14
Vegetated – HLC	78	20	17-25
Urban - BGT	351	14	12-17
Urban - HLC	79	28	24-34
Log-rank test (p)		5e-07	

* HLC= Human Landing Catch; BGT= BG-Sentinel Trapping

Fig S1. Histogram and Shapiro-wilk test to assess normality of the longevity data.

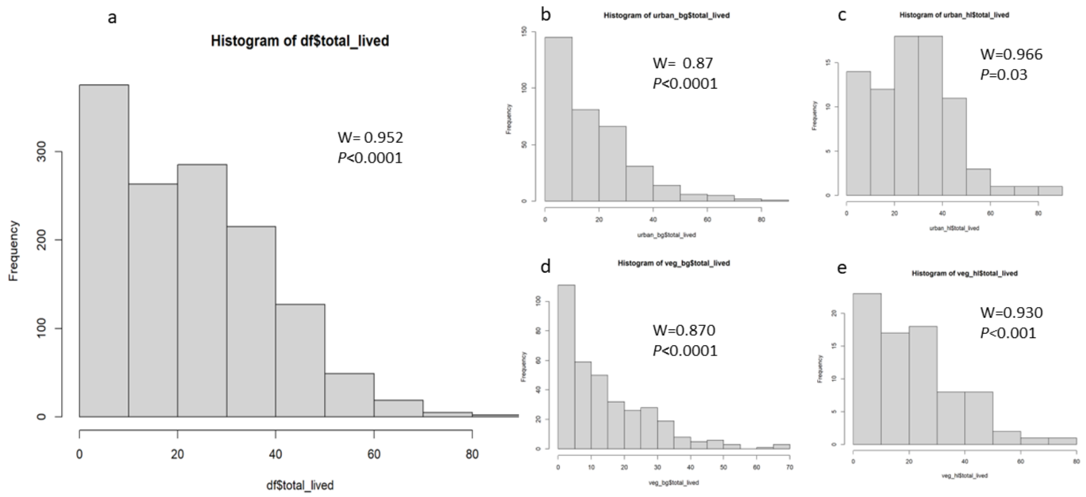

Fig S2. Correlation plot of the variables included in the Cox Mixed models.

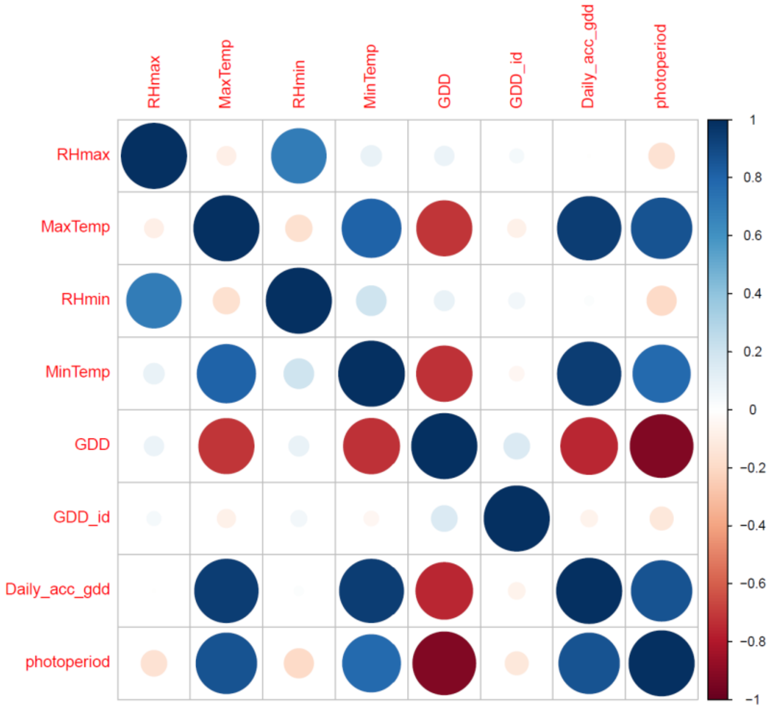

Fig S3. Kruskall-Wallis and Dunn test with Bonferroni correction, showing the differences in post-capture longevity through the months of the experiment in the urban area

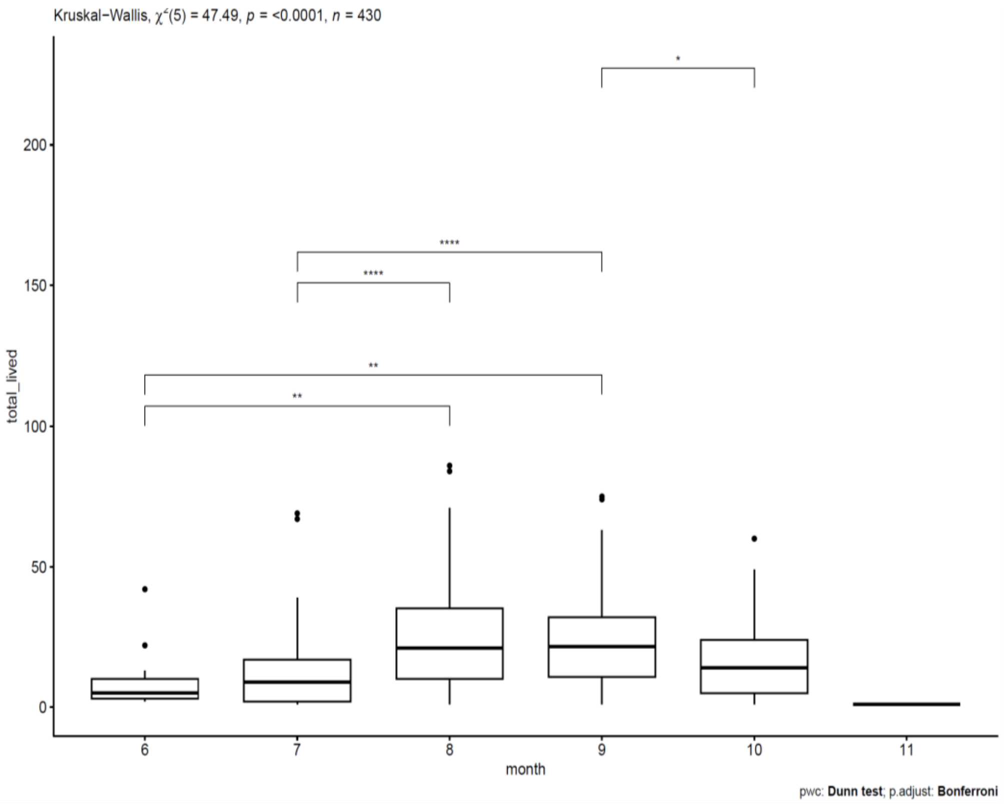

Fig S4. Kruskall-Wallis and Dunn test with Bonferroni correction, showing the differences in post-capture longevity through the months of the experiment in the vegetated area

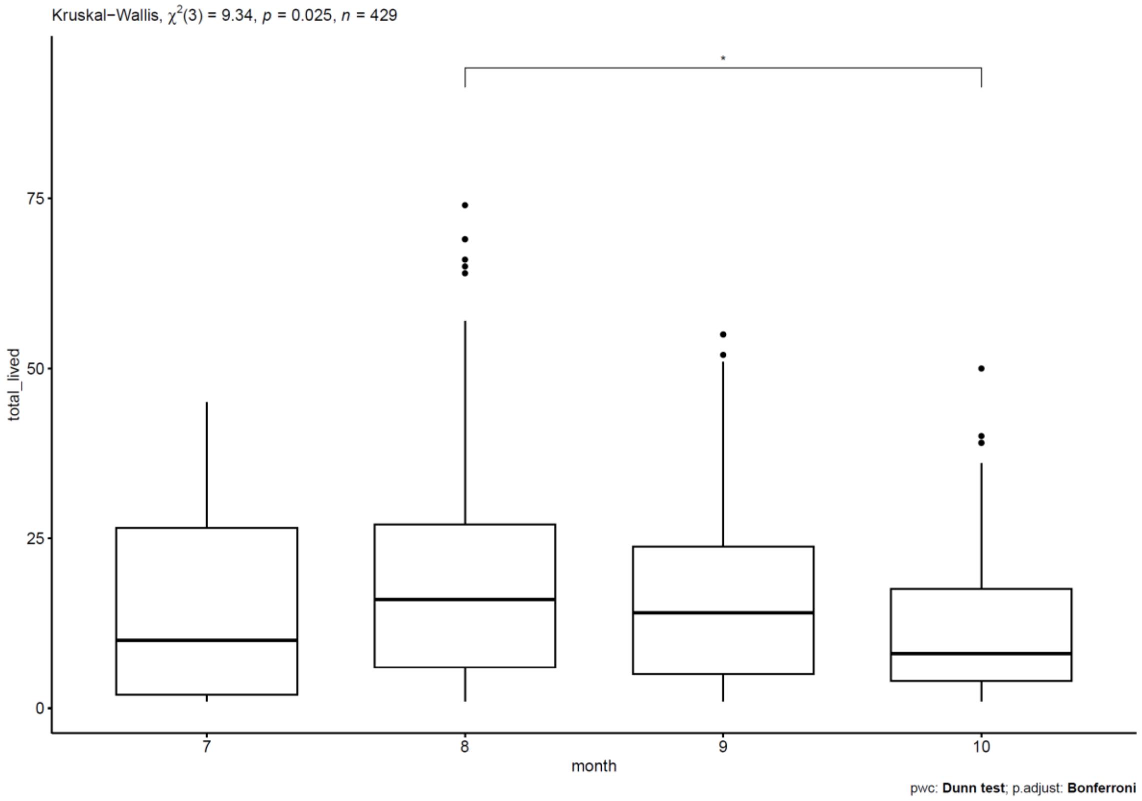

Fig S5. Significance test for the differences between the meteorological conditions of Temperature (a) and Relative Humidity (b) in both field sites.

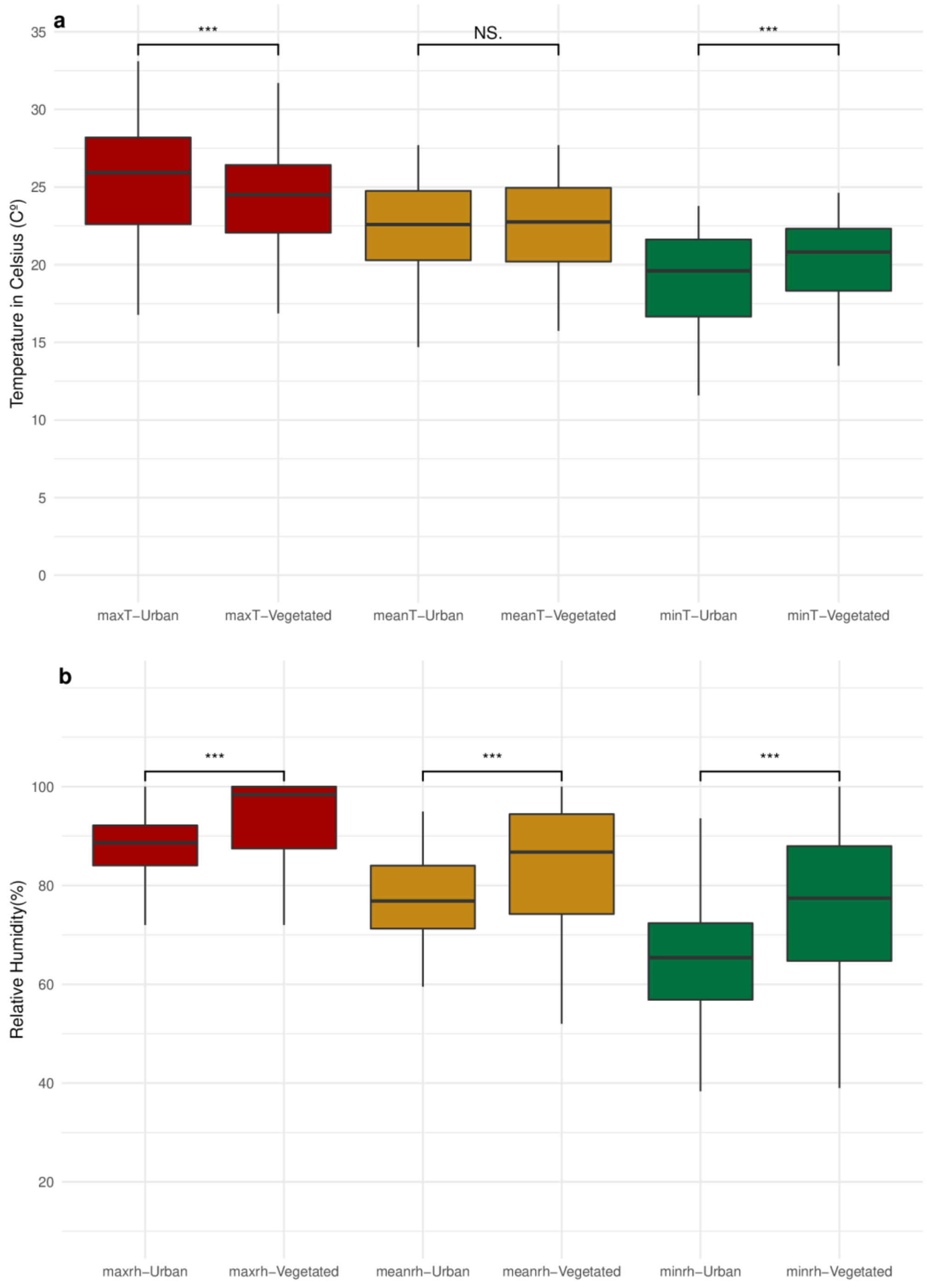

Supplement: Supplementary file 1 — Additional file 1: Table S1. Kaplan-Meier analysis, comparing survival curves of differenr locations (field and labpratory), capture methods (human landing catches, BG-Sentinel trapping) and their combinations. Figure S1. Histogram and Shapiro-Wilk test to assess normality of the longevity data. Figure S2. Correlation plot of the variables included in the Cox mixed models. Figure S3. Kruskall-Wallis and Dunn test with Bonferroni correction, showing the differences in post-capture longevity through the months of the experiment in the urban area. Figure S4. Kruskall-Wallis and Dunn test with Bonferroni correction, showing the differences in post-capture longevity through the months of the experiment in the vegetated area. Figure S5. Significance test for the differences between the meteorological conditions of (a) temperature and (b) relative humidity (b) both field sites. [file 13071_2023_5961_MOESM1_ESM.pdf]
